# Prevalence of Mechanical and Biological Complications of Dental Implants in Diabetic Versus Non-diabetic Patients: An Institutional Database Study

**DOI:** 10.7759/cureus.109505

**Published:** 2026-05-23

**Authors:** Jay Gohil, Iram Saba, Ramanjeet K Grover, Haifa Beefathimathul, Akhilesh Malethia, Tushar Ranjan

**Affiliations:** 1 Department of Prosthodontics, K.M. Shah Dental College and Hospital, Sumandeep Vidyapeeth (Deemed to be University), Vadodara, IND; 2 Department of Dentistry, Shri Siddhi Vinayak Medical College and Hospital, Sambhal, IND; 3 Department of Prosthodontics, Maharaja Ganga Singh Dental College and Research Centre, Sri Ganganagar, IND; 4 Department of Prosthetic Dental Sciences, College of Dentistry, Qassim University, Buraydah, SAU; 5 Department of Prosthodontics, Surendera Dental College and Research Institute, Sri Ganganagar, IND; 6 Department of Prosthodontics, Buddha Institute of Dental Sciences and Hospital, Patna, IND

**Keywords:** dental implants, diabetes mellitus, glycemic control, implant complications, peri-implantitis

## Abstract

Introduction: Diabetes mellitus may adversely influence peri-implant tissue health and outcomes. The aim of this study was to evaluate and compare the prevalence of mechanical and biological complications associated with dental implants in diabetic and non-diabetic patients and to assess the influence of glycemic control.

Materials and Methods: This retrospective observational study was conducted using the institutional database records from January 2019 to December 2023. A total of 168 patient's database were included and equally divided into diabetic (n = 84) and non-diabetic (n = 84) groups based on their medical history and glycated hemoglobin (HbA1c) levels. Inclusion criteria comprised adult patients (≥18 years) with single endosseous implants and a minimum loading period of 12 months. The assessed mechanical complications included abutment screw loosening, prosthetic screw fracture, veneer fracture, framework fracture, and occlusal wear. Biological complications included bleeding on probing, suppuration, probing depth ≥6 mm, radiographic bone loss ≥2 mm, and peri-implantitis. Patients with diabetes were further stratified into controlled (HbA1c ≤7%) and uncontrolled (HbA1c >7%) groups. Statistical analysis was performed, with p < 0.05 considered statistically significant.

Results: The mean age was significantly higher in diabetic patients (58.4 ± 9.7 years) compared to non-diabetic patients (54.1 ± 10.3 years; p = 0.003). Biological complications were significantly more prevalent in diabetic patients, including bleeding on probing (odds ratio (OR): 2.94; p < 0.001), suppuration (OR: 3.33; p = 0.007), probing depth greater than or equal to 6 millimeters (OR: 3.17; p = 0.002), radiographic bone loss ≥2 millimeters (OR: 3.27; p = 0.003), and peri-implantitis (OR: 3.73; p = 0.002). Biological complications were observed in 54 (64.3%) diabetic patients compared with 31 (36.9%) non-diabetic patients (p < 0.001). Mechanical complications were more frequent in patients with diabetes, with abutment screw loosening being significantly higher (p = 0.026), while other mechanical complications were not statistically significant. Among diabetic patients, those with uncontrolled glycemic status showed significantly higher biological complications than control patients, including peri-implantitis (p = 0.028).

Conclusion: Diabetes mellitus, particularly poor glycemic control, significantly increases the risk of biological complications in dental implants, whereas mechanical complications are less affected. Careful patient selection, metabolic control, and rigorous maintenance are essential for achieving optimal implant outcomes.

## Introduction

Dental implants have become a predictable and widely accepted treatment modality for the replacement of missing teeth, demonstrating high survival and success rates over long-term follow-up [1]. However, implant outcomes are influenced by multiple patient-related and systemic factors that may compromise osseointegration and peri-implant tissue stability [2,3]. Among these, diabetes mellitus, particularly type 2 diabetes mellitus (T2DM), has emerged as a critical modifier of implant prognosis owing to its well-documented effects on wound healing, immune response, and bone metabolism [4].

Chronic hyperglycemia is associated with impaired collagen synthesis, reduced osteoblastic activity, and increased levels of inflammatory mediators, all of which may predispose individuals to peri-implant tissue breakdown and delayed healing. Consequently, diabetic patients are considered at a higher risk of biological complications, such as peri-implant mucositis and peri-implantitis [5,6]. In addition to biological factors, diabetes may also indirectly contribute to mechanical complications through altered occlusal dynamics, compromised bone support, and changes in prosthetic load distribution [7].

Despite growing evidence, the literature remains inconsistent regarding the extent to which diabetes affects implant success, particularly when glycemic control is considered. Many studies have focused primarily on implant survival rates, often overlooking the broader spectrum of mechanical and biological complications that significantly impact long-term clinical outcomes and patient satisfaction [8,9]. Furthermore, limited data exist from large institutional databases that allow the comprehensive evaluation of these complications in real-world clinical settings.

Therefore, this study is aimed to evaluate and compare the prevalence of mechanical and biological complications associated with dental implants in patients with and without diabetes using institutional database records. The objectives were to (i) assess the frequency and types of mechanical complications, (ii) evaluate the prevalence of biological complications, including peri-implantitis, and (iii) analyze the influence of glycemic control on complication rates within the diabetic cohort.

## Materials and methods

This study was designed as a retrospective observational institutional database analysis conducted at the Department of Prosthodontics, Buddha Institute of Dental Sciences and Hospital, Patna, India. The study evaluated implant records over a five-year period from January 2019 to December 2023, providing a comprehensive longitudinal assessment of implant-related complications. This retrospective design enabled the inclusion of real-world clinical data while minimizing intervention-related bias.

Prior to data collection, ethical approval was obtained from the Institutional Ethical Committee. Owing to the retrospective nature of the study and the use of anonymized patient records, a waiver of individual informed consent was granted. All procedures adhered to the institutional ethical standards and complied with the principles outlined in the Declaration of Helsinki for research involving human participants.

Inclusion criteria

**Figure 1:**
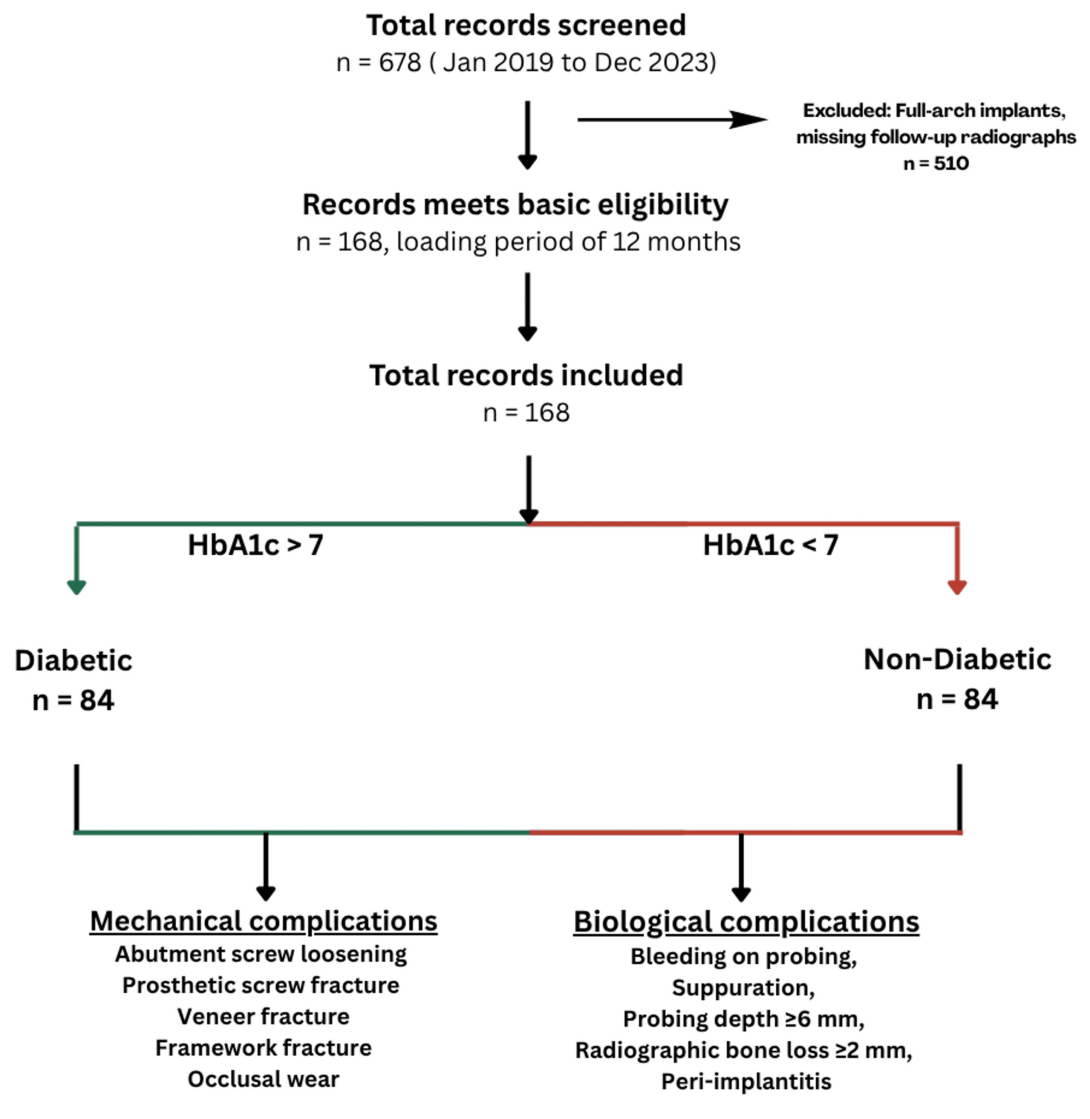
Study flowchart.

A total of 678 implant case records were initially screened from the institutional database. Following the application of predefined inclusion and exclusion criteria, 168 patients were selected and equally allocated into two groups, diabetic (n = 84) and non-diabetic (n = 84), based on documented medical history and glycated hemoglobin (HbA1c) levels. The institutional database was screened for records of adult patients (≥18 years) who received at least one single endosseous dental implant with a minimum functional loading period of 12 months. Eligible records contained a complete medical history and a documented glycated HbA1c value within six months prior to implant placement. Patients were included if they were either systemically healthy (non‑diabetic) or had a confirmed diagnosis of diabetes mellitus (type 2), regardless of glycemic control status (Figure 1).

Exclusion criteria

Records were excluded if the patient had multiple or full‑arch implants, missing follow‑up radiographs or clinical examination data at the 12‑month loading visit, or a lack of documented HbA1c levels. Patients with uncontrolled systemic diseases other than diabetes (e.g., osteoporosis, autoimmune disorders, and active malignancy), history of head and neck radiotherapy, heavy smoking (>10 cigarettes/day), or pregnancy during the observation period were also excluded. Incomplete demographic or complication records led to automatic exclusion.

Sample size estimation

The required sample size was estimated using G*Power software (version 3.1, Heinrich Heine University, Düsseldorf, Germany). Based on published literature indicating that the risk of peri‑implantitis is approximately 50% higher in diabetic patients compared to non‑diabetic patients [10], a two‑tailed significance level (α) of 0.05 and a statistical power of 80% were applied. Assuming equal group sizes and no design effect (i.e., simple random sampling), the initial calculation yielded approximately 75 participants per group. Accounting for an anticipated 10-12% rate of incomplete or missing records, the sample size was adjusted upward to 84 participants per group, resulting in a total study sample of 168 participants.

Outcome measurement

Outcome variables were categorized into mechanical and biological complications. Mechanical complications include abutment screw loosening, prosthetic screw fracture, veneer fracture (minor and catastrophic), framework fracture, and occlusal wear. Biological complications included bleeding on probing, suppuration, probing depth ≥6 mm, radiographic bone loss ≥2 mm, and peri-implantitis, as defined by the 2017 World Workshop criteria [11]. For patients with diabetes, subgroup analysis was performed based on glycemic control (HbA1c ≤7% and >7%). Data, including demographic variables, systemic status, implant characteristics, and complication outcomes, were systematically extracted using a standardized data collection form. Radiographic assessment was performed using standardized periapical radiographs with consistent reference points.

Statistical analysis

Statistical analysis was conducted IBM SPSS Statistics for Windows, Version 26.0 (Released 2019; IBM Corp., Armonk, New York). Descriptive statistics were calculated for all the variables. Data were confirmed for normal distribution with Shapiro-Wilk test. Continuous variables were expressed as mean ± standard deviation and compared using the independent samples t-test, whereas categorical variables were analyzed using the chi-square test or Fisher’s exact test. Binary logistic analysis was performed for group and subgroup to calculate the odds ratios (OR) with 95% confidence intervals (CI). Statistical significance was set at p < 0.05.

## Results

Table 1 shows the demographic and clinical characteristics of the study population. The mean age was significantly higher in the diabetic group (58.4 ± 9.7 years) compared to the non-diabetic group (54.1 ± 10.3 years; p = 0.003). The sex distribution was comparable (p = 0.752). A higher proportion of smokers was observed among patients with diabetes than among those without diabetes, although the difference was not statistically significant (p = 0.063). Mean HbA1c levels were significantly higher in diabetics (7.8 ± 1.3%) than in those without non-diabetics (5.3 ± 0.4%; p < 0.001). Follow-up duration was similar between groups (38.6 ± 14.2 vs. 37.9 ± 13.8 months; p = 0.728).

**Table 1:** Demographic and clinical characteristics of study participants (n = 168). Values are presented as mean ± standard deviation (SD) or number (percentage). Independent samples t-test (t) was used for continuous variables and chi-square test (χ²) for categorical variables. *Statistically significant (p < 0.05).

Variable characteristic		Diabetic (n = 84)	Non-diabetic (n = 84)	Test value (df)	p-value
Age (years)	Mean ± SD	58.4 ± 9.7	54.1 ± 10.3	t(166) = 2.79	0.003*
Sex, n (%)	Male	49 (58.3)	51 (60.7)	χ²(1) = 0.10	0.752
	Female	35 (41.7)	33 (39.3)		
Smoking status, n (%)	Smoker	29 (34.5)	18 (21.4)	χ²(1) = 3.57	0.063
	Non-smoker	55 (65.5)	66 (78.6)		
Glycated hemoglobin level, n (%)	Controlled (≤7.0%)	38 (45.2)	Not applicable	Not applicable	Not applicable
	Uncontrolled (>7.0%)	46 (54.8)	Not applicable		
Glycated hemoglobin level	Mean HbA1c ± SD	7.8 ± 1.3	5.3 ± 0.4	t(166) = 16.85	< 0.001*
Follow-up duration	Mean (months) ± SD	38.6 ± 14.2	37.9 ± 13.8	t(166) = 0.32	0.728

Table 2 summarizes the prevalence of the mechanical complications. Abutment screw loosening was significantly more common in patients with diabetes than in those without diabetes (OR: 2.49; 95% CI: 1.11-5.58; p = 0.026). Prosthetic screw fractures occurred in 12 (14.3%) patients with diabetes and in 6 (7.1%) patients without diabetes (p = 0.132). Minor veneer fractures were observed in 18 (21.4%) and 14 (16.7%) patients (p = 0.443), while catastrophic veneer fractures occurred in seven (8.3%) vs. three (3.6%) patients (p = 0.205). Framework fractures and occlusal wear were also higher in patients with diabetes, but the difference was not statistically significant.

**Table 2:** Prevalence of mechanical complications by diabetic status. Values are presented as number (percentage). Odds ratios and 95% confidence intervals were calculated to quantify the strength and direction of association between categories. *Statistically significant (p < 0.05).

Mechanical complication	Diabetic (n = 84), n (%)	Non-diabetic (n = 84), n (%)	Total (n = 168), n (%)	Odds ratio (95% confidence interval)	p-value
Abutment screw loosening	23 (27.4)	11 (13.1)	34 (20.2)	2.49 (1.11-5.58)	0.026*
Prosthetic screw fracture	12 (14.3)	6 (7.1)	18 (10.7)	2.17 (0.79-5.98)	0.132
Veneer fracture (minor)	18 (21.4)	14 (16.7)	32 (19.0)	1.36 (0.62-2.97)	0.443
Veneer fracture (catastrophic)	7 (8.3)	3 (3.6)	10 (6.0)	2.44 (0.61-9.73)	0.205
Framework fracture	5 (6.0)	2 (2.4)	7 (4.2)	2.59 (0.49-13.8)	0.261
Occlusal wear (significant)	14 (16.7)	9 (10.7)	23 (13.7)	1.67 (0.67-4.16)	0.271

Biological complications were significantly more prevalent in patients with diabetes (Table 3). Bleeding on probing was recorded in 51 (60.7%) diabetic patients compared to 29 (34.5%) non-diabetic patients (OR: 2.94; p < 0.001). Suppuration occurred in 22 (26.2%) vs. 8 (9.5%) (OR: 3.33; p = 0.007), probing depth ≥6 mm in 31 (36.9%) vs. 13 (15.5%) (OR: 3.17; p = 0.002), and radiographic bone loss ≥2 mm in 28 (33.3%) vs. 11 (13.1%) (OR: 3.27; p = 0.003). Peri-implantitis was diagnosed in 26 (31.0%) diabetic patients compared to 9 (10.7%) non-diabetic patients (OR: 3.73; p = 0.002). Overall, biological complications were present in 54 (64.3%) diabetic and 31 (36.9%) non-diabetic patients (OR: 3.08; p < 0.001).

**Table 3:** Prevalence of biological complications by diabetic status. Values are presented as number (percentage). Odds ratios and 95% confidence intervals were calculated to quantify the strength and direction of association between categories. *Statistically significant (p < 0.05).

Biological complication	Diabetic (n = 84), n (%)	Non-diabetic (n = 84), n (%)	Total (n = 168), n (%)	Odds ratio (95% confidence interval)	p-value
Bleeding on probing (BOP)	51 (60.7)	29 (34.5)	80 (47.6)	2.94 (1.54-5.62)	< 0.001*
Suppuration	22 (26.2)	8 (9.5)	30 (17.9)	3.33 (1.37-8.09)	0.007*
Probing depth ≥6 mm	31 (36.9)	13 (15.5)	44 (26.2)	3.17 (1.50-6.72)	0.002*
Radiographic bone loss ≥2 mm	28 (33.3)	11 (13.1)	39 (23.2)	3.27 (1.49-7.17)	0.003*
Peri-implantitis	26 (31.0)	9 (10.7)	35 (20.8)	3.73 (1.60-8.72)	0.002*
Any biological complication	54 (64.3)	31 (36.9)	85 (50.6)	3.08 (1.60-5.92)	< 0.001*

Table 4 presents subgroup analysis within diabetics based on glycemic control. Uncontrolled diabetics (HbA1c >7%) showed higher rates of biological complications compared to controlled diabetics (HbA1c ≤7%), including bleeding on probing (71.7% vs. 47.4%; OR: 2.82; p = 0.029), probing depth ≥6 mm (47.8% vs. 23.7%; OR: 2.97; p = 0.028), radiographic bone loss ≥2 mm (43.5% vs. 21.1%; OR: 2.92; p = 0.033), and peri-implantitis (41.3% vs. 18.4%; OR: 3.12; p = 0.028). Suppuration (34.8% vs. 15.8%; p = 0.056) and overall biological complications (73.9% vs. 52.6%; p = 0.051) showed a strong trend but did not reach statistical significance. Mechanical complication (abutment screw loosening: 32.6% vs. 21.1%; p = 0.251) did not differ significantly between subgroups.

**Table 4:** Complication rates in diabetic group stratified by glycemic control (HbA1c). Values are presented as number (percentage). Odds ratios and 95% confidence intervals were calculated to quantify the strength and direction of association between categories. Controlled diabetes: glycated hemoglobin ≤7.0%, uncontrolled diabetes: glycated hemoglobin >7.0%. *Statistically significant (p < 0.05).

Complication	Controlled diabetes (n = 38), n (%)	Uncontrolled diabetes (n = 46), n (%)	Odds ratio (95% confidence interval	p-value
Mechanical complications				
Abutment screw loosening	8 (21.1)	15 (32.6)	1.80 (0.66-4.92)	0.251
Biological complications				
Bleeding on probing	18 (47.4)	33 (71.7)	2.82 (1.11-7.20)	0.029*
Suppuration	6 (15.8)	16 (34.8)	2.81 (0.97-8.16)	0.056
Probing depth ≥6 mm	9 (23.7)	22 (47.8)	2.97 (1.12-7.87)	0.028*
Radiographic bone loss ≥2 mm	8 (21.1)	20 (43.5)	2.92 (1.09-7.82)	0.033*
Peri-implantitis	7 (18.4)	19 (41.3)	3.12 (1.13-8.61)	0.028*
Any biological complication	20 (52.6)	34 (73.9)	2.57 (0.99-6.64)	0.051

## Discussion

This retrospective institutional database study evaluated the prevalence of mechanical and biological complications associated with dental implants in diabetic and non-diabetic patients, with additional stratification based on glycemic control. The findings demonstrate that diabetes mellitus, particularly when poorly controlled, is significantly associated with an increased risk of biological complications, whereas its influence on mechanical complications appears comparatively limited.

In this study, biological complications, including bleeding on probing, suppuration, increased probing depth, radiographic bone loss, and peri-implantitis, were significantly more prevalent in patients with diabetes than in those without diabetes. These findings are consistent with those of previous studies that identified diabetes as a major risk factor for peri-implant disease. For instance, Monje et al. [10] reported a higher prevalence of peri-implantitis among diabetic individuals, particularly those with poor glycemic control, attributing this to an impaired host immune response and chronic inflammatory burden. Similarly, a systematic review by Chrcanovic et al. [12] demonstrated that hyperglycemia adversely affected peri-implant tissue stability and increased marginal bone loss. Similar results were reported by Li et al. [13].

The biological plausibility of these findings lies in the pathophysiological mechanism of diabetes. Chronic hyperglycemia leads to the formation of advanced glycation end-products, which disrupt collagen turnover and impair wound healing [6]. Additionally, altered neutrophil function and increased pro-inflammatory cytokines contribute to a heightened inflammatory state, facilitating peri-implant tissue breakdown [14]. The significantly higher odds ratios observed in this study further reinforce the strength of this association.

In contrast, mechanical complications were only modestly higher in patients with diabetes, with abutment screw loosening being the only significant finding. Other complications, such as prosthetic screw fracture, veneer fracture, framework fracture, and occlusal wear, did not differ significantly between the groups. These findings are in agreement with prior literature, suggesting that mechanical complications are more influenced by prosthetic design, occlusal factors, and implant positioning than by systemic conditions alone [15]. Goodacre et al. [16] emphasized that prosthetic complications are multifactorial and often independent of systemic disease status.

A key strength of this study is the subgroup analysis based on glycemic control, which revealed that uncontrolled diabetics (HbA1c >7%) experienced significantly higher rates of biological complications than controlled diabetics. Notably, peri-implantitis and probing depth of ≥6 mm were markedly increased in the uncontrolled group. These findings corroborate those of Oates et al. [17], who reported that well-controlled diabetic patients exhibit implant outcomes comparable to those of non-diabetic patients, whereas poor glycemic control significantly compromises peri-implant health. This underscores the importance of metabolic control as a modifiable risk factor for implant dentistry.

Clinical implications

From a clinical perspective, these findings highlight the need for careful patient selection and risk assessment in patients with diabetes undergoing implant therapy. Preoperative evaluation of the glycemic status using HbA1c levels should be considered as a standard protocol. Patients with uncontrolled diabetes should be counseled regarding the increased risk of peri-implant complications, and elective implant placement should be deferred until adequate glycemic control is achieved.

Furthermore, enhanced maintenance protocols, including more frequent recall visits, strict plaque control measures, and early intervention strategies, are essential for diabetic patients. Clinicians should also consider modifying treatment planning by optimizing implant positioning, ensuring an appropriate prosthetic design, and minimizing occlusal overload to reduce both biological and mechanical risks. Interdisciplinary collaboration with physicians for glycemic management is crucial for improving the overall treatment outcomes.

Limitations

Despite its strengths, this study had several limitations. The retrospective design inherently introduces the possibility of selection bias and limits the control of confounding variables. Although efforts were made to exclude cases with incomplete records, variations in clinical documentation and follow-up protocols may have influenced outcome assessment. Additionally, factors such as oral hygiene status, implant surface characteristics, loading protocols, and clinician experience were not fully standardized or accounted for, which could have affected the complication rates. Although adequately powered, the sample size was derived from a single-institutional database, which may limit the generalizability of the findings to broader populations. Moreover, this study relied on HbA1c values recorded at baseline without accounting for fluctuations in glycemic control over time. Longitudinal prospective studies with standardized protocols are required to validate these findings.

## Conclusions

Within the limitations of this retrospective study, diabetes mellitus was significantly associated with a higher prevalence of biological complications in dental implants, particularly peri-implant inflammation, bone loss, and peri-implantitis. Poor glycemic control further exacerbates these risks, highlighting its critical role in implant prognoses. In contrast, mechanical complications showed only a limited association with diabetes status, with abutment screw loosening being the only significant finding. These results emphasize the importance of preoperative glycemic assessment, strict metabolic control, and tailored maintenance protocols to optimize implant outcomes in patients with diabetes and improve long-term peri-implant health.
